# Supplementary material for: The Systems Biology Research Tool: evolvable open-source software
Source: BMC Syst Biol. 2008 Jun 29;2:55. doi: 10.1186/1752-0509-2-55 (PMC2446383; doi:10.1186/1752-0509-2-55)
Supplement: Additional file 1 — SBRT Archive. An archive of the current version of the Systems Biology Research Tool. [file 1752-0509-2-55-S1.zip › sbrt-1.4.0/doc/developers_guide/api/sbrt/kernel/math/algebra/MatrixTransposer.html]

MatrixTransposer


|  |  |  |
| --- | --- | --- |
| **Overview** \| **Package** \| **Class** \| **Use** \| **Tree** \| **Deprecated** \| **Index** \| **Help** | |  |
| **PREV CLASS**   **NEXT CLASS** | **FRAMES**    **NO FRAMES**     **All Classes** |  |
| SUMMARY: NESTED \| FIELD \| CONSTR \| METHOD | DETAIL: FIELD \| CONSTR \| METHOD |  |


---


## sbrt.kernel.math.algebra Class MatrixTransposer

```
java.lang.Object
  sbrt.kernel.math.algebra.MatrixTransposer
```

---

``` public class MatrixTransposer extends java.lang.Object ```

This class is used to transpose matrices.

**Author:**
:   This class was written and documented by Jeremiah Wright
    while in the Wagner lab.

---

**Constructor Summary**

`MatrixTransposer()`


**Method Summary**

`<R,C,E> void`

`transpose(Matrix<R,C,E> matrix,
MatrixBuilder<? super C,? super R,E> emptyMatrix)`
  
          Fills `emptyMatrix` with the transpose of `matrix`.

**Methods inherited from class java.lang.Object**

`clone, equals, finalize, getClass, hashCode, notify, notifyAll, toString, wait, wait, wait`

**Constructor Detail**

### MatrixTransposer

```
public MatrixTransposer()
```


**Method Detail**

### transpose

```
public <R,C,E> void transpose(Matrix<R,C,E> matrix,
                              MatrixBuilder<? super C,? super R,E> emptyMatrix)
```

:   Fills `emptyMatrix` with the transpose of `matrix`.

    :   **Type Parameters:**: `R` - the row index type.: `C` - the column index type.: `E` - the element or value type. **Parameters:**: `matrix` - the matrix for which a transpose will be created.: `emptyMatrix` - the empty matrix where the tranpose will be stored. **Throws:**: `java.lang.IllegalArgumentException` - if `emptyMatrix` is not empty.: `java.lang.NullPointerException` - if either matrix is `null`.


---


|  |  |  |
| --- | --- | --- |
| **Overview** \| **Package** \| **Class** \| **Use** \| **Tree** \| **Deprecated** \| **Index** \| **Help** | |  |
| **PREV CLASS**   **NEXT CLASS** | **FRAMES**    **NO FRAMES**     **All Classes** |  |
| SUMMARY: NESTED \| FIELD \| CONSTR \| METHOD | DETAIL: FIELD \| CONSTR \| METHOD |  |


---
